# Hepatocellular Carcinoma in Patients with Chronic Hepatitis C and Liver Cirrhosis Treated with DAA: A Focused Review

**DOI:** 10.3390/jcm14051505

**Published:** 2025-02-24

**Authors:** Sandica Bucurica, Andreea-Simona Nancoff, Raluca Ioana Marin, Carmen Monica Preda

**Affiliations:** 1 Department of Gastroenterology, Carol Davila University of Medicine and Pharmacy, 020021 Bucharest, Romania; bucuricasandy@yahoo.com (S.B.); andreea-simona.nancoff@rez.umfcd.ro (A.-S.N.); 2 Department of Gastroenterology, University Emergency Central Military Hospital “Dr. Carol Davila”, 024185 Bucharest, Romania; 3 Department of Gastroenterology, Fundeni Clinic Institute, 022328 Bucharest, Romania; ralucaionamarin@yahoo.com

**Keywords:** hepatocellular carcinoma, chronic hepatitis C, liver cirrhosis, direct antiviral agents

## Abstract

**Background/Objectives**: The issue of HCC recurrence in patients with liver cirrhosis and chronic HCV infection after DAA treatment as well as the issue of de novo HCC in individuals with chronic HCV hepatitis treated with DAA is of great importance. In this review, the two important aspects are discussed and, finally, an algorithm for approaching the patient with HCC and chronic HCV infection is proposed. **Methods**: A literature search of the two databases (PubMed and Scopus) was conducted using the terms ‘chronic hepatitis C’ and/or ‘liver cirrhosis’ and ‘hepatocellular carcinoma’, from database inception to December 2024. **Results**: Thirty-one studies have examined the risk of HCC recurrence. Most of these studies conclude that DAA treatment reduces the risk of HCC recurrence compared to patients who did not receive DAA. There are considerable differences across various world regions. These variations may arise from: differences in genotypes, baseline characteristics of the populations, variability in DAA treatment protocols, and differences in follow-up intervals. Eleven studies that investigated the issue of de novo HCC after DAA were reviewed, of which two included historical cohorts of untreated patients. **Conclusions**: The conclusion is that these patients present a low or equal risk of HCC incidence compared to untreated patients, and the risk factors for HCC are: lower platelet number, impaired liver function, nonresponse to DAA. Most patients with chronic hepatitis C and HCC should receive DAAs, except for those in BCLC stage D, but we must emphasize that timing of intervention is crucial and it is very important to evaluate possible drug interactions.

## 1. Introduction

Hepatocellular carcinoma (HCC) ranks as the sixth leading cause of cancer-related deaths worldwide, with mortality rates expected to escalate in the coming decade [1]. Incidence rates of HCC exhibit significant variability across nations, being associated with the prevalence of distinct risk factors within different geographical regions [2,3]. Common risk factors include viral hepatitis infections (hepatitis B and C), non-alcoholic fatty liver disease (NAFLD), and alcoholic liver disease, all of which culminate in liver fibrosis and cirrhosis, ultimately predisposing individuals to HCC [2]. Like most cancers, HCC develops when combined environmental and genetic factors disrupt normal cell growth [4]. The progression to HCC is typically a multi-step and complex process involving various molecular pathways [5]. Regenerative hepatic nodules associated with cirrhosis create favorable local conditions in which normal hepatic cells become dysplastic and eventually cancerous [6]. Furthermore, both local and systemic inflammation—mediated by inflammatory pathways, cytokines, chemokines, and growth factors—alter hepatocyte function, accelerating the onset and progression of HCC [6,7]. Treatment resistance and high tumor recurrence rates present formidable challenges in managing HCC [2]. Surgical interventions, including liver resection, serve as primary treatment options for early-stage hepatocellular carcinoma (HCC), aiming to eliminate localized tumors [8]. However, due to the heterogeneous nature of HCC, a personalized treatment strategy is essential for advanced stages. This approach should integrate locoregional therapies, systemic agents, and immunotherapies to optimize patient outcomes [2,3]. HCV represents a substantial global health challenge, with an estimated 180 million individuals affected worldwide, with higher rates in regions such as Africa, the Eastern Mediterranean, South-East Asia, and the West Pacific and lower prevalence rates in North America, Northern and Western Europe, and Australia [9,10,11]. The virus is characterized by seven major genotypes, each with distinct subtypes, exclusively pathogenic to humans [9,12,13]. Chronic HCV infection, which develops in 75–80% of cases, poses a significant clinical burden due to its potential to progress to complications such as liver cirrhosis, hepatocellular carcinoma (HCC), and necessitating liver transplantation [14,15]. Diagnosis of acute HCV infection remains challenging due to asymptomatic or mild symptoms [9]. Historically, HCV treatment relied on interferon-based therapy, which was limited in efficacy and associated with significant side effects [15,16]. Direct eradication of HCV with DAAs is believed to decrease the risk of HCC development, highlighting the importance of effective antiviral therapy in reducing cancer risk [16,17]. DAAs represent a significant advancement in HCV therapy, with sustained virological response (SVR) rates consistently exceeding 90% for genotype 1, even in a cirrhotic state [16,18,19]. However, recent studies have emerged showing a steady rise of HCC recurrence among patients previously treated with DAAs for HCV [20,21]. One possible explanation is that direct-acting antiviral (DAA) treatment induces a pronounced viral clearance of HCV, thereby disrupting the equilibrium of immune cell responses and the balance of cytokine pathways [22], thus accelerating the process of angiogenesis [23]. A potential relationship between these has yet to be fully established, but new research has been conducted in this area of interest [20,21,24].

Through this review, we aim to identify the potential risk factors associated with the recurrence of HCC in patients who have previously undergone treatment. Additionally, we emphasize the need for guidance for clinicians facing the challenge of deciding whether to initiate direct-acting antiviral DAA therapy in patients with chronic HCV. By synthesizing the latest evidence and weighing the potential benefits and risks, we aim to inform evidence-based decision-making and optimize patient outcomes in this challenging clinical scenario.

## 2. Materials and Methods

This narrative review examines the multifaceted impact of direct-acting antiviral (DAA) therapy in patients with chronic hepatitis C and liver cirrhosis, focusing on three critical aspects: (1) the potential risk of HCC recurrence in patients with treated chronic HCV infection, liver cirrhosis, and a history of prior HCC treatment; (2) the risk of de novo HCC development in patients with chronic hepatitis C and liver cirrhosis who have received DAA therapy; and (3) the suitability of DAA treatment for all patients with chronic hepatitis C, liver cirrhosis, and a history of HCC.

**Figure 1:**
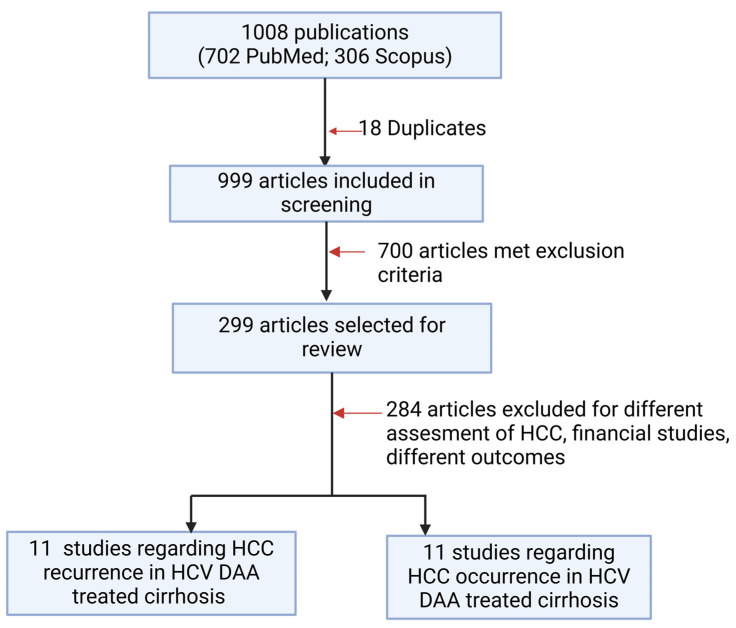
Study’s chart flow.

A literature search of the two databases (PubMed and Scopus) was conducted using the terms ‘chronic hepatitis C’ and/or ‘liver cirrhosis’ and ‘hepatocellular carcinoma, from database inception to December 2024. Two authors thoroughly assessed the key articles identified in the literature review (S.B. and A.S.N). Next, the selected titles, abstracts, and studies were evaluated, and biases were solved through discussions among the authors. The inclusion criteria consisted of clinical studies conducted up to December 2024, human population studies, type of antiviral therapy administered, recurrence rate of HCC, follow-up interval, as well as the time from the last treatment of HCC up to the initiation of DAA treatment, sustained virological response rate per protocol, possible predictor factors (Figure 1).

We excluded systematic reviews or other types of reviews, studies conducted on non-human populations, and studies that did not focus on DAA treatment and its association with HCC recurrence (Figure 1). This method aimed to offer a nuanced comprehension of the current literature and its relevance to clinical practice.

## 3. Results

### 3.1. Risk of HCC Recurrence in Patients with Chronic Hepatitis C and Liver Cirrhosis and Treated HCC That Received DAA

While direct-acting antiviral (DAA) therapy has represented a revolutionary advancement in the treatment of hepatitis C virus (HCV), with a remarkable sustained virological response (SVR) rate and demonstrating beneficial effects on liver function enhancement and reduction of cirrhosis-related complications, its impact on the development of de novo or recurrent HCC remains an important topic [25,26,27,28]. Early data suggested a high recurrence rate of HCC in patients who previously had a complete response to loco-regional HCC treatment and were subsequently treated with DAAs [23,29,30,31]. As mentioned, this recurrence can be attributed to various molecular and immunological changes occurring after DAA treatment [32]. It is widely speculated that the rapid decrease in NK cell activation and cytotoxicity after therapy can promote HCC recurrence [33]. NK cells are innate immune cells providing a first-line defense mechanism against pathogens [34]. In addition to this role, they exert anti-tumor effects by recognizing and neutralizing cancerous cells [35]. In some cases, repeated exposure to malignant cells and tumor microenvironment can alter their physiological activity, thus decreasing cytotoxicity agents in cancerous cells [36]. As demonstrated by Zhang X et al. (2022), direct-acting antiviral (DAA) treatment did not affect the total number of NK cells; however, the antiviral therapy did alter the subsets of these immune cells, as well as the cytokines produced, particularly IFN-γ and TNF-α. They also showed that, although the number of NK cells was partially restored in the first week of DAA treatment, their anti-cancerous function significantly decreased 12 weeks following the completion of DAA treatment [37]. Other theories that have been postulated are those related to MAIT cell (mucosal-associated invariant T cell) dysfunction or the “normalized” liver microenvironment that may support HCC progression by disrupting immunological balances in the liver [15,38]. In chronic inflammation, the liver’s immune microenvironment, which consists of distinctive but complex cellular pathways, becomes overwhelmed [39]. In this scenario, different oncogenetic factors can escape from the surveillance of local immune cells that, under normal circumstances, act as carcinogen blockers, promoting HCC development [40]. HCC’s molecular mechanisms of onset, progression, and recurrence remain topics of interest for many researchers but still appear not fully uncovered. Therefore, future studies need to be directed towards this area.

Eleven original studies are summarized in our comprehensive review, all of which approach the following details: the number of patients included, the combination of direct-acting antiviral therapy, SVR rate, HCC recurrence rate, an interval of time between HCC eradication and DAA initiation, the interval time of HCC recurrence, as well as the main findings of the study and possible predictive factors for HCC recurrence. Some systematic reviews have been conducted to address this issue, yet they have collectively determined that there is insufficient evidence to suggest an elevated risk of HCC in patients undergoing DAA therapy for HCV; emerging evidence suggests a significant decrease in the risk of HCC occurrence or recurrence following DAA treatment, with reported reductions of up to 71% [16,25,41,42].

**Table 1:** HCC recurrence after DAA therapy.

Study	No of Patients	DAA Therapy	Recurrence Rate	Control Group	Median Follow-Up Interval (min–max)	Median Time from HCC Eradication to DAA Therapy	Time of Recurrence Median (Range Months)	SVR Rate per Protocol	Conclusions	Predictive Factors for Recurrence
Preda, C.M. et al. (2018) [16]	24	3D + RBV	5.5 vs. 24.6% (*p* = 0.044)	Yes	44 months	≥6 months	4 months (3–6 months)	87.5%	Decrease of recurrence and improvement of survival rates.	Not identified.
Pop, C.S. et al. (2020) [25]	28	LDV/SOF ± RBV	44.4%	No	20 months (5–24).	≥3 months	6–12 months	66.7%	High recurrence rate among DAA treated patients with Decompensated cirrhosis.	Impaired liver function. Low platelet number.
Idilman, R. et al. (2019) [26]	200	LDV/SOF ± RBV	9%	No	22.1 months (15.7–30.3)	14 months	6 months	98%	Decreased risk of de novo HCC among DAA treated atients. Higher frequency HCC rate among those who had a short period of time between diagnosis and DAA initiation.	Not identified
Zanetto, A. et al. (2017) [29]	23	LDV/SOF SOF/DCV SMV/SOF SOF +RBV	12.5% vs. 8.3% (*p* = 0.6)	Yes	10 months (6–19) vs. 7 months (5–19)	N/A *	7 months vs. 12 months	100%	DAAs does not appear to increase the risk of patients dropping out LT waiting list due to the progression of HCC.	Not identified.
Yoshimasu, Y. et al. (2019) [43]	234	DCV + ASV SOF/LDV 3D ELB + GZR SOF + RBV	13% vs. 35.4% vs. 35.4%	No	21 months (6–38)	N/A *	N/A *	91.5%	Decreased occurrence and recurrence after DAA treatment.	AFP levels AFP- L3% APRI value Albumin level
Conti, F. et al. (2016) [44]	344	SOF/SMV 3D SOF + RBV SOF/DCV SOF/LDV DCV/SMV	7.6%	No	24 weeks	376 days (45–2706 days)	N/A *	91%	DAA treatment does not decrease the risk of HCC occurrence or recurrence.	Not identified.
ANRS [45]	267 79 314	SOF + RBV + PEGIFN SOF + DCV ± RBV SOF + LDV ± RBV SOF + RBV SOF + SMV ± RBV 3D ± RBV SMV + DCV SOF + LDV + RBV SOF + DCV	12.7% vs. 7.7% vs. 2.2%	No	20.2 months vs. 21.3 months (13.0–33.5) vs. N/A	N/A * vs. 16.5 months (12.7–32.2) vs. 67 months (7–127)	N/A *	91.9% vs. 100% vs. 96.8%	No association between DAA treatments and the risk of HCC recurrence after the implementation of curative procedures.	Not identified.
Zavaglia, C. et al. (2016) [46]	31	SOF/LDV ± RBV SIM/SOF SOF/DCV ± RBV 3D SOF/RBV	3.2%	No	8 months (5–10.9)	19.3 months (12.6–3.9)	N/A *	100%	No association between DAA treatments and the risk of HCC recurrence after the implementation of curative procedures.	Not identified.
Virlogeux, V. et al. (2017) [47]	68	SOF SOF/DCV SOF/LDV SOF/SIM 3D ±RBV	1.7/100 person-months	Yes	>12 months	7.2 months	17.4 months (5.3–44.4) vs. 10.1 months (2.3–59.4	96%	Recurrence rate of HCC was lower after DAA treatment.	Not identified.
Reig, M. et al. (2016) [48]	53	SOF/LDV 3D SOF/SMV SOF/DCV SMV/DCV ±RBV	27.6%	No	5.7 months (0.4–14.6)	11.2 months (3.6–23.3)	N/A *	97.5%	High recurrence rate of HCC among DAA treated patients, especially when there is a short period of time between HCC treatment and DAA initiation.	Not identified.
Tahata, Y. et al. (2021) [49]	388	ASV/DCV SOF/RBV LDV/SOF 3D EBR/GZR	19.2% vs. 32.3% vs. 43.0%	No	26.9 months	11.5 months (2.2–83.7)	28.1 months (1.3–53.4)	N/A *	DAA treatment was Associated with lower risk of HCC recurrence and better survival. No significant difference between IFN and DAA therapies regarding HCC recurrence patterns was found.	Not identified

N/A *: Not applicable.

Table 1 summarizes the main features of DAA therapy in both cirrhotic and non-cirrhotic patients and its outcomes regarding HCC association. Various interferon-free therapies were analyzed to establish the association between the eradication of HCV infection and HCC, both in cirrhotic and non-cirrhotic patients, as well as in patients with a previous history of HCC and those with no history of this disease. In almost all studies we analyzed, we can see that the SVR rate was similar, with values ranging from 91% to 100%, aligning with data from the literature [29,46,47,50]. Several differences were observed when evaluating the risk of HCC’s recurrence following DAA treatment. In most studies, the risk of HCC recurrence appeared to decrease after DAA therapy. However, some studies presented results that contradicted this finding. Researchers conducted by Pop CS et al., Conti F et al., and Reig M et al. indicated a marked recurrence after DAA treatment. In some cases, this was associated with a short period between the treatment of HCC and initiation of DAA treatment [25,44,48,50]. Similar results were also reported by Warzyszyńska K et al. as the relapse of HCC was almost two times accelerated in their DAA-analyzed group (265 days after surgery in the DAA group vs. 532 days in the NDAA (non-DAA) group (*p* = 0.033)) [51].

**Figure 2:**
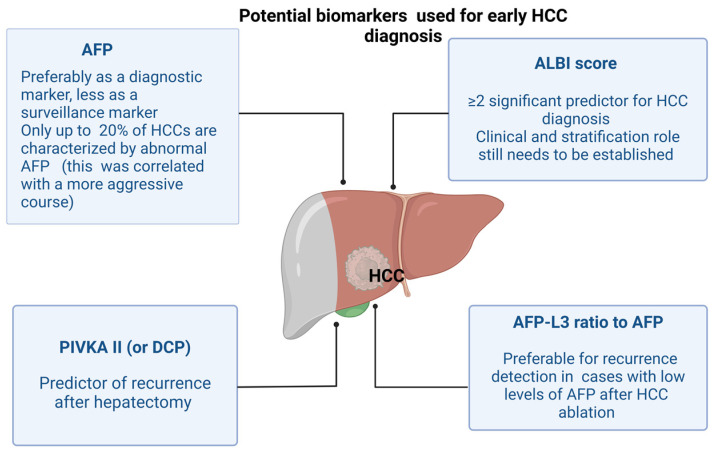
Potential serological test used for early HCC diagnosis. (AFP—alpha-fetoprotein, AFP-L3 glycosylated AFP (L3 fraction) to total AFP, PIVKA II—prothrombin induced by vitamin K absence II, DCP—des-gamma-carboxy prothrombin, ALBI score—Albumin-Bilirubin score) [56,57,61].

Several factors have been identified as potential contributors to HCC risk. Specific biomarkers, such as alpha-fetoprotein levels (AFP), are widely used for HCC surveillance and to appreciate the risk of recurrence [52]. Furthermore, recent studies have identified additional potential biological markers that may predict the odds of recurrence [52,53]. In recent years, the ALBI score has been recognized as a reliable early predictor of HCC recurrence, with a value of ≥2 proving to be a significant predictor [54,55]. PIVKA-II (prothrombin induced by vitamin K absence-II (PIVKA-II), a relatively new marker, was reported by Wang LR. et al. to be an independent indicator of HCC’s early recurrence after hepatectomy [56,57] (Figure 2).

Other predictive parameters include high-sensitivity C-reactive protein (hs-CRP) and albumin ratio, as Ren Y. et al. proved to be an independent marker of the reappearance and progression of HCC [58], programmed cell death protein-1 (PD-1), microRNAs, and proteins in urinary exosomes, Model for End-Stage Liver Disease (MELD) scores, presence of fibrosis or cirrhosis, the time elapsed since curative cancer treatment, history of multiple treatments, and previous recurrences are also associated with HCC reappearance [27,59,60].

In a prospective study by Preda CM et al., 24 patients diagnosed with HCV-associated cirrhosis and previously treated HCC were administrated reimbursed 3D + RBV for 12 weeks. The study investigates critical considerations in patients with previously treated HCC in HCV-associated cirrhosis: whether, when, and how to administer DAA therapy and the impact of 3D + RBV on overall survival in these patients [25]. Inclusion criteria consisted of no previous HCC recurrence 6 months after the last procedure of HCC treatment, as seen by imagistic findings (contrast-enhanced CT or contrast-enhanced MRI). The authors managed to prove the following results: (1) SVR was achieved in 21 out of 24 patients with a rate of 87.5% (2) Patients who underwent DAA treatment were compared with a historical cohort of subjects who underwent the same procedures for the treatment of HCC but did not receive DAA, HCC recurrence rates were as follows: TACE—transarterial Chemoembolization: 37.5% vs. 100%, *p* = 0.026; RFA—radiofrequency ablation group: 3 (21.4%) vs. 12 (85.7%) *p* = 0.002); (3) positive outcomes including improved survival without recurrence and enhanced overall survival in patients with compensated HCV-related cirrhosis and HCV-related HCC. The primary limitation of this study is the small patient cohort, with only 24 patients. In the existing literature, several studies have explored HCC recurrence. Still, few have included a control group of age, gender, and BCLC—Barcelona Clinic Liver Cancer staging-matched patients without antiviral therapy post-initial HCC treatment, mirroring this study [16,62].

Another cohort study, originating from Italy by Conti F. et al., enrolled 285 patients, among whom 59 were chronic HCV-infected, cirrhotic patients with a history of HCC [44]. Subjects were monitored for up to 24 weeks after DAA treatment, and the following results were noted: de novo occurrence of HCC was identified in 26 out of 285 (7.6%) patients. However, a notably high rate of recurrence of previously successfully treated HCC was observed in 17 (29%) of the 59 patients [44]. Reig M. et al. also reported similar results, which align with other findings in the literature [27]. Regarding the HCC recurrence rate per 100 patient years, various studies on HCC relapse post-treatment report disparate percentages ranging from 2.2% to 47% [45,48,50,63]. However, several other publications have reported no increase in the HCC relapse rate [47,49]. Zavaglia C et al. contradicted Reig and Conti’s findings. They proposed that their longer interval between complete tumor eradication and antiviral therapy (median 19 months compared to 11 months in Reig’s study) might explain, at least partially, the differing outcomes. The short interval presented by Reig and colleagues might be associated with undetected radiological recurrence or remaining tumoral tissue [46]. Yet, in other studies, the longer the interval between the last treatment procedure for HCC and the initiation of DAA therapy was associated with higher recurrence rates [16,45]. Virlogeux V. et al. included 68 patients with HCV infection in remission and previous HCC diagnosis, treated or non-treated with direct-acting antivirals (DAAs); 96% achieved SVR, resembling data from the literature [29,46,47,50]. The main results consist of a decrease in HCC recurrence among patients with a history of this disease as well as HCV infection who received DAA treatment. (*p* = 0.008). Similar results were also found in the existing publications. However, as a retrospective cohort study, there was no randomization of patients; there were incomplete histological data on HCC grade for all patients, with only 44% (N = 32) having this information, so the severity of HCC was assessed based on the size and number of nodules. This cohort consisted solely of cirrhotic patients, who often had additional comorbidities. Given the clinical significance of these findings, they need to be validated in a larger, more diverse HCV-HCC population, especially concerning disease severity [47].

Extensive cohort studies from various countries (France, Italy, the US, Japan, [45]) showed a positive relation regarding HCC recurrence after DAA treatment, with rates ranging from 1.4% to 12.7%. This further establishes the role of antiviral therapy in lowering the risk of developing HCC. Similar results were also reported by Lui et al., as the risk of HCC recurrence in the treated group was 64 times lower than in the untreated group [64]. However, these results highlight the considerable differences observed across various world regions. These variations may arise from factors such as differences in genotypes, baseline characteristics of the studied populations, variability in DAA treatment protocols, exclusion of suspicious lesions before treatment with DAA (presence of non-characterizable hepatic nodules), and differences in follow-up intervals.

### 3.2. Risk of HCC Occurrence in Patients with Chronic Hepatitis C and Liver Cirrhosis Who Received DAA

In a prospective study by Sangiovanni et al., 1285 cirrhotic patients were enrolled, either without or with a history of HCC. The aim was to evaluate the recurrence and the occurrence of HCC in DAA-treated patients with HCV-related cirrhosis. Notably, the highest HCC incidence occurred within the first year of antiviral treatment initiation, aligning with findings from other studies indicating potential DAA treatment-related progression of premalignant nodules or clinically undetected liver cell cancer clones to overt HCC [44,65]. However, these studies suggest increased HCC incidence post-DAA treatment. Non-cardio-selective beta-blockers, ascites, albumin levels, total number of platelets, INR values, and levels of AFP were independent risk factors for HCC occurrence in cirrhotic patients with no history of liver cancer. The theory that DAA therapy may enhance HCC development links the early onset of HCC incidence to alterations in the liver microenvironment involved in cancer immune surveillance [50,65,66]. This is believed to result from the rapid suppression of HCV and related cell signals. As we mentioned before, recent evidence indicates that DAAs induce upregulation of VEGF expression in the liver, which promotes HCC occurrence/recurrence in susceptible patients, particularly those with advanced fibrosis or cirrhosis, who already exhibit abnormal activation of neo-angiogenic pathways like angiopoietin-2 in their liver tissue [67].

Elevated AFP levels are well-recognized markers that identify high-risk populations. However, in the Sangiovanni A et al. study, only 2 out of 35 patients with AFP > 100 ng/mL at enrollment developed HCC during follow-up. This suggests that HCV-related chronic inflammation might play a role in the development of HCC rather than undetected cancer clones, contributing to the elevated value of this tumor marker in these patients [66].

Marino Z et al. conducted a retrospective multicenter study centered on cirrhotic patients undergoing treatment with direct-acting antivirals up to December 2016. Clinical and radiological data were gathered before initiating antiviral therapy and during follow-up, with additional data collected at the onset of HCC development. The diagnosis of HCC underwent centralized validation, and its incidence was quantified as HCC cases per 100 person-years [68]. 1123 patients were included (60.6% males, 83.8% Child-Pugh A), and 95.2% achieved a sustained virologic response. The median time of follow-up was 19.6 months. Seventy-two patients developed HCC within a median of 10.3 months after starting antiviral treatment. HCC incidence was 3.73 HCC/100 person-years (95% CI 2.96–4.70). Baseline liver function, alcohol intake, and hepatic decompensation were associated with a higher risk of HCC. The relative risk was significantly increased in patients with non-characterized nodules at baseline 2.83 (95% CI 1.55–5.16) vs. absence of non-characterized nodules [68] (Table 2). Marino Z et al. reported a rate of HCC occurrence similar to that reported by Sangiovanni A et al. 3.1/100 patient-years. This large cohort included 1123 patients with high rates of SVR per protocol (95.2%). Unlike Perrella A et al., the risk of HCC recurrence was assessed in a shorter period after HCV infection treatment. Hence, they managed to show that the risk of early HCC recurrence did not improve after DAA treatment. Therefore, a thorough follow-up period is still essential for detecting liver cancer in the early stages. Some of the predictive factors reported by the authors were similar to those mentioned in the literature [21,66,69].

In their study, Perrella A. et al. analyzed the risk of developing HCC in a cohort of 306 patients for 48 months. They reported an occurrence rate of 6.55%, which aligns with other findings in the literature. Some of the predictive factors for HCC de novo appearance were reported to be CTP B stage (*p* = 0.001), presence of diabetes (*p* = 0.007), cirrhosis (*p* = 0.002), and the liver stiffness value (*p* = 0.0001) [21].

The observation that 75% of patients who developed de novo HCC had BCLC 0/A HCC (predominantly with a single nodule) aligns with previous reports indicating 71% of Milan-in HCC cases in patients under surveillance. This finding is consistent with recent studies by Calvaruso et al. and Mariño et al., which challenge the hypothesis that DAAs may enhance the onset of clinically aggressive HCC [66,68,70,72].

The risk of occurrence and recurrence of hepatocellular carcinoma (HCC) among cirrhotic patients following antiviral therapy with a combination of LDV/SOF ± RBV was evaluated by Pop CS et al. and Idilman et al. The two studies analyzed similar baseline characteristics, follow-up periods, and antiviral treatments but reported differing outcomes. In their multicenter retrospective study, including only patients with decompensated cirrhosis, Pop CS et al. observed a 4.7% HCC occurrence rate during a median follow-up interval of 20 months, and the most important predictive factors for the occurrence of HCC are: poor liver function and low platelet count. Based on their findings, it appears that DAA therapy in cirrhotic patients neither elevates nor reduces the incidence of HCC. While they observed a lower SVR in patients who developed de novo HCC compared to those unaffected by HCC, this disparity did not attain statistical significance. However, it’s essential to note that this situation could be subject to bias, particularly due to the limited number of patients in the occurrence group [25,26]. In contrast, Idilman et al. reported a 98% SVR rate and a notably low recurrence rate of HCC (9%). Furthermore, only one patient without a history of HCC exhibited de novo occurrence [26]. Overall, DAA treatment among CHC patients was associated with better outcomes and improvement of liver function, as well as with a lower incidence of HCC among the subjects.

Table 2 presents the most important studies in the literature dedicated to the issue of HCC after DAA treatment. Of these, only 2 include a historical control cohort: the study by Cheung et al. and the study by Kilany et al. [65,71]. Cheung et al. conclude that the prevalence of HCC in patients with liver cirrhosis and chronic hepatitis C treated with DAAs is similar to the prevalence rate in untreated patients, while Kilany et al. demonstrate a significant reduction in the occurrence of hepatocellular carcinoma in people who received DAAs.

### 3.3. Should All Patients with Chronic Hepatitis C Liver Cirrhosis and HCC Be Treated with DAA?

Hepatitis C virus (HCV) infection is a major contributor to liver cirrhosis and hepatocellular carcinoma (HCC), frequently necessitating liver transplantation. The advent of direct-acting antiviral agents (DAAs) has revolutionized HCV therapy, yielding high rates of sustained virological response (>98%). We need to discuss the usefulness of antiviral treatment in patients with chronic viral hepatitis C separately and successfully treated and inactive hepatocellular carcinoma and patients with active hepatocellular carcinoma (untreatable or with active recurrence).

The first category of patients, those with hepatocellular carcinoma treated with curative intent (through resection procedures or RFA) and those with inactive HCC after chemoembolization, clearly benefit from treatment with direct antivirals. Most data in the literature suggest that antiviral treatment reduces the frequency of recurrence, according to the studies discussed previously [16,25,26,29,43,44,45,46,47,48,49].

The timing of DAA treatment after curative HCC is crucial. Waiting several months allows the immune response to keep microscopic HCC clones under control. DAA treatment is not an emergency after curative treatment of hepatocellular carcinoma: most data in the literature suggest waiting 4–6 months after curative procedure, during which time at least 2 multi-phase CT or MRI examinations should be performed to confirm the absence of recurrence [61,73]. The reason why we recommend at least 2 CT or MRI imaging examinations within this 4–6 month interval from the curative procedure is that data from the literature suggests that a single imaging examination has a sensitivity of only 40–50% for lesions smaller than 1 cm and 60–70% for those with a diameter between 1 and 2 cm [61].

In contrast, for patients with “active” and intractable HCC, the data are controversial. We defined “active” HCC as a liver nodule in which there is viable tumor tissue: the presence of viable tumor tissue is defined according to the EASL clinical practice guidelines on the management of HCC 2025, i.e., rim and non-rim arterial hyperenhancement, peripheral and non peripheral washout (in the portal venous or delayed phases on CT and MRI using extracellular contrast agents or gadobenate dimeglumine, or in the portal venous phase only with MRI using gadoxetic acid. The arguments in favor of treating these patients should be discussed individually, depending primarily on their prognosis: as we well know, both the EASL and AGA guidelines do not recommend treatment with direct antivirals if the person concerned has severe comorbidities with a serious impact on survival (including patients with active intractable hepatocellular carcinoma) [73,74]. Also, treatment must be discussed on a personalized and individualized basis and depending on the risk of drug interactions (hep-drug-interactions). In our clinical practice, we do not recommend DAA treatment in patients with intermediate or advanced active HCC and cirrhosis in whom we anticipate a survival of less than 2 years. Furthermore, there are no studies in the literature to evaluate the survival benefit and whether antiviral treatment is cost-effective.

We must take into account the fact that certain studies in the literature suggest a statistically signifficant lower SVR rate in patients with HCC and chronic HCV hepatitis compared to those without HCC: In the meta-analysis published by Fanpu Ji et al. the SVR rate differed significantly between patients with HCC and those without HCC: 89.6% vs. 93.3%, *p* = 0.0012, and this difference seems to come mainly from the very different SVR rates between patients with “active” HCC versus those with imaging evidence of “inactive” HCC: 73.1% vs. 92.6% (*p* = 0.002) [60].

Ji F’s data are also confirmed by the meta-analysis by He S, who found an SVR of 88.2% in patients with HCC and chronic HCV hepatitis compared with 92.4% in patients without HCC (*p* < 0.001). However, it should be noted that He S and co-authors do not report differences between the SVR achieved in patients with cirrhosis and those with cirrhosis and HCC (89.1% vs. 89.4%), the conclusion of this analysis being that the different SVR rates are explained mainly by liver function [75].

Based on recent research findings, the question of whether all patients with chronic hepatitis C, liver cirrhosis, and HCC should receive DAA remains very complex. Figure 2 illustrates how the patient with HCC and chronic HCV infection should be approached, in light of the latest data in the literature.

**Figure 3:**
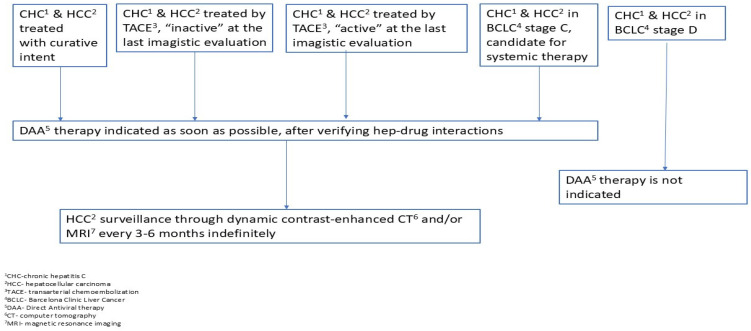
Proposed algorithm for the management of patients with HCC and chronic viral hepatitis C.

In any case, the timing of DAA treatment in patients with HCC and cirrhosis and chronic HCV infection is extremely important. If the patient can benefit from curative intervention for hepatocellular carcinoma (surgical resection or radiofrequency ablation), antiviral treatment will be performed after this intervention (at an interval of 3–6 months, after imaging has documented that there is no residual tumor tissue). In the case where the individual with HCC and liver cirrhosis and HCV infection is listed for liver transplantation, the timing of administering antiviral treatment depends on the waiting time on the transplant list and liver function: if the waiting time is too long and the patient’s risk of deterioration is high, then antiviral treatment should be administered before transplantation [73] (Figure 3).

Clinicians will need to consider the following parameters when prescribing antiviral treatment with DAAs in people with active HCC and hepatitis C viral cirrhosis: (1) HCC stage and the possibility of completely treating HCC, (2) liver function, (3) survival prognosis (4) patient agreement regarding treatment timing and therapeutic regimen.

Regarding the safety and efficacy of DAA therapy, it has been proven to be a potent treatment against HVC infection throughout the years. Doing so significantly reduced the financial burden associated with complications arising from chronic infection and HCC development [62,71,76].

## 4. Conclusions

While DAAs have transformed HCV treatment by achieving high SVR rates, concerns have arisen regarding their potential impact on HCC occurrence or recurrence.

As a perspective, HCC’s molecular mechanisms of onset, progression, and recurrence remain topics of interest for many researchers but still appear not fully uncovered. Therefore, future studies need to be directed towards this area.

New extensive cohort studies show that in most cases, HCC recurrence decreased after DAA treatment, especially when the antiviral therapy was started within the optimal time interval of at least 4–6 months from the last curative procedure for HCC. Most studies sustain these results. Regarding de novo HCC occurrence, some current evidence that suggests DAA therapy does not significantly alter the incidence of HCC in cirrhotic patients. However, we agree that individual patient factors, such as comorbidities and baseline liver function, should be carefully considered when determining the appropriateness of DAA treatment for those with HCV-related cirrhosis and HCC.

Longer-term follow-up studies of these patients are certainly necessary to more accurately assess the dimensions of the problem and to confirm how the risk of de novo HCC in this population of cirrhotics evolves over time, as well as the risk of HCC recurrence.

## Figures and Tables

**Table 2:** The HCC occurrence after DAA therapy in patients with HCV cirrhosis.

Study	No of Patients	DAA Therapy	Occu Rrence Rate	Con Trol Group	Median Follow-Up Interval (min–max)	Time of Occurrence Median (Range Months)	SVR Rate per Proto Col	Conclusions	Predictive Factors for HCC Ocurrence
Ogata, F. et al. (2017) [19]	1170	DCV/ASV SOF/LDV 3D	2.3%	No	24 months	16 months	91%	Eradication of HCV RNA by direct-acting antiviral regimens might reduce the risk of HCC	Albumin α-fetoprotein levels
Perrella, A. et al. (2024) [21]	306	SOF/RBV SOF/LDV SOF/DCV SOF/SMV 3D	6.55%	No	48 months	N/A	100%	DAA therapy was not associated with a lower risk of late HCC development.	Presence of cirrhosis Liver stiffness Diabetes Child-Turcotte -Pugh B
Pop, C.S. et al. (2020) [25]	331	SOF/LDV	4.7%	No	20 months (5 ÷ 24)	6 months (3 ÷ 18)	84.5%	The occurrence rate of HCC was 4.7% in a median follow-up period of 20 months as expected in this population with decompensated cirrhosis.	lower platelet number impaired liver function
Yoshimasu, Y. et al. (2019) [43]	211 53% with cirrhosis	DCV/ASV SOF/LDV 3D SOF/RBV EBR/GZR	1%	No	24 months (6 ÷ 40)	24 months	91.5%	The HCC occurrence rate after DAA treatment was very low	AFP level AFP-L3%
Conti, F. et al. (2016) [44]	285	SOF/LDV 3D EBR/GZR	3.16%	No	6 months	6 months	91%	DAA-induced resolution of HCV infection does not seem to reduce occurrence of HCC	Child-Pugh Class B, more severe liver fibrosis, lower platelet count
Cheung, M. et al. (2016) [65]	406	SOF/LDV 3D EBR/GZR	5%	Yes	15 months	N/A	78.1%	No significant difference in liver cancer incidence for treated patients vs. untreated patients.	Child-Pugh Class B
Sangiovanni, A. et al. (2020) [66]	1285	SOF/LDV SOF/DCV SOF/RBV 3D SOF/SMV EBR/GZR SOF/VEL, SOF/LDV + DCV SOF/DSV	4.1%	Yes	17 months	10 months (1–27) for de novo of HCC	96%	DAA therapy was not associated with rapid onset of HCC development, but it reported a possible time-dependent association between HCC occurrence and previous undefined liver nodules detected before DAA initiation.	Ascites alpha-feto protein values undefined liver nodules
Marino, Z. et al. (2019) [68]	1123	SOF/LDV 3D SOF SOF/SMV SOF/DCV SMV/DCV	3.73 HCC/100 person-years	No	19.6 months	10.3 months	95.2%	DAA therapy was not associated with a lower risk of early HCC development.	History of alcohol consumption Previous UNMNs detected before DAA initiation. Liver stiffness
Tada, T. et al. (2021) [69]	355	DCV/ASV SOF/LDV 3D SOF/RBV EBR/GZR GLE/PIB SOF/VEL	4%	No	36.8 months (18.3–47.8)	N/A	N/A	Transient elastography can be used to predict the incidence of HCC occurrence. Advanced hepatic fibrosis is a associated with a higher risk of HCC development, but no association was found between this and advanced hepatic steatosis	Hepatic fibrosis
Calvaruso, V. et al. (2018) [70]	2249	SOF/LDV 3D EBR/GZR	3.5%	No	14 months (6 ÷ 24)	9.8 months (2 ÷ 22)	95.2%	SVR to DAA treatment decreased the incidence of HCC over a mean follow-up of 14 months.	Low albumin Level Low platelet Count Absence of SVR
Kilany, S. et al. (2021) [71]	1630	SOF/LDV 3D SOF SOF/SMV SOF/DCV SMV/DCV	4.8%	Yes	23 months (1 ÷ 43)	N/A	N/A	Incidence of HCC significantly lower in patients with HCV-related advanced fibrosis and cirrhosis treated with DAAs than in a historical cohort of untreated patients.	Decompen- sated cirrhosis, baseline AFP ≥ 10 ng/mL, diabetes, Nonresponse to DAA

UNMNs—undefined/non-malignant nodules; SOF—sofosbuvir; SOF/VEL—sofosbuvir/velpatasvir; SOF/RBV—sofosbuvir/ribavirin; SOF/LDV—sofosbuvir/ledipasvir; SOF/DCV—sofosbuvir/daclatasvir, SOF/SMV—sofosbuvir/simeprevir; 3D—ombitasvir/paritaprevir/ritonavir + dasabuvir; RBV—ribavirin; EBR/GZR—elbasvir/grazoprevir; GLE/PIB—glecaprevir/pibrentasvir. N/A—not applicable.

## Abbreviations

The following abbreviations are used in this manuscript:

DAA	Direct Antiviral Agents
HCC	Hepatocellular carcinoma
HCV	Hepatitis C virus
BCLC	Barcelona Clinic Liver Cancer
NAFLD	non-alcoholic fatty liver disease
HCV	Hepatitis C virus
SVR	sustained virological response
NK	Natural Killer
IFN	Interferon
TNF-α	Tumor necrosis factor-α
MAIT	mucosal-associated invariant T cell
SOF	sofosbuvir
SOF/VEL	sofosbuvir/velpatasvir
SOF/RBV	sofosbuvir/ribavirin
SOF/LDV	sofosbuvir/ledipasvir
SOF/DCV	sofosbuvir/daclatasvir
SOF/SMV	sofosbuvir/simeprevir
3D	ombitasvir/paritaprevir/ritonavir + dasabuvir
RBV	ribavirin
EBR/GZR	elbasvir/grazoprevir
GLE/PIB	glecaprevir/pibrentasvir
AFP	alpha-fetoprotein levels
PIVKA	prothrombin induced by vitamin K absence
CT	Computed tomography
MRI	Magnetic Resonance Imaging
TACE	transarterial Chemoembolization
RFA	radiofrequency ablation
EASL	European Association for the Study of the Liver
AGA	American Gastroenterological Association
